# Open Rigid Internal Fixation of Low-Neck Condylar Fractures of the Mandible: Mechanical Comparison of 16 Plate Designs

**DOI:** 10.3390/ma13081953

**Published:** 2020-04-22

**Authors:** Marcin Kozakiewicz, Rafał Zieliński, Bartłomiej Konieczny, Michał Krasowski, Jakub Okulski

**Affiliations:** 1 Department of Maxillofacial Surgery, Medical University of Lodz, 1st Gen. J. Hallera Pl., 90-647 Lodz, Poland; bkost@op.pl (R.Z.); jakub.okulski@gmail.com (J.O.); 2 Material Science Laboratory, Medical University of Lodz, 251st Pomorska, 92-213 Lodz, Poland; bartlomiej.konieczny@umed.lodz.pl (B.K.); michal.krasowski@gmail.com (M.K.)

**Keywords:** mandibular condyle, low-neck fracture, surgical treatment, ORIF, dedicated plates, mechanical comparison

## Abstract

Background: In the literature, no information on plates for low-neck mandibular condylar osteosynthesis can be found, despite the fact that 30 plate designs have already been published. The aim of this study was to compare any dedicated plates for possible use in low-neck condylar fracture osteosynthesis. Methods: The force required for 1-mm displacement of the fixed fracture fragments and incidents of screw loosening were recorded on polyurethane mandibles among 16 designs of titanium plates fixed by 6-mm screws in a 2.0 system. Results: Double-straight plate fixation was the mechanical gold standard (15.2 ± 3.5 N), followed by A-shape Condylar Plates (14.9 ± 2.1 N), X-shape Condylar Plates (14.2 ± 1.3 N) and Auto Repositioning Plates (11.8 ± 2.4 N). Screw loosening was uncommon, as a minimum of three screws were placed into the condylar part. Fewer screws were lost from the ramus part of the fixation if the plate was attached to the condylar part by three screws. Often, the stability of the ramus screws was lost when there were only two fixing screws in the condyle (*p* < 0.001). Conclusions: It is advisable to consider the mechanical advantages as one decides which plate to choose for open rigid internal fixation in low-neck condylar fractures, or to only be aware of the significant differences in mobility within the fracture line after fixation with different dedicated plates.

## 1. Introduction

Condylar fractures of the mandible are one of the most frequent injuries observed in the facial skeleton [1]. Therapy is difficult, and a large number of patients do not achieve correct bite conditions after treatment. Biomechanical evaluation shows that the treatment of mandibular neck fractures cannot resolve malocclusion outcomes, as the fixation is not sufficiently rigid [2]. Therefore, currently, open reduction and rigid internal plate fixation (ORIF) has become the first choice of therapy [3].

Recently, 30 dedicated titanium plates were compared in basal condylar fracture osteosynthesis [4]. Only four plate designs out of the thirty designs were shown to withstand screw pull-out and displacing force. Then, challenging high-neck fracture fixations were also tested [5], but there are no studies in the literature that show which type of plate fixation is superior for the treatment of low-neck mandibular condyle fractures. Some authors admit that the application of double-plain plates is the most rigid, but it can be very demanding and is not always possible [6].

It has been difficult to indicate significant differences among monocortical plating techniques [7]. Currently, a single plain miniplate is used during endoscopic intraoral fixations. As Haug et al. noticed many years ago, four different plain plates could reach similar mechanical results; however, the explanation of this phenomenon is not easy. Regardless of the almost abandoned technique of condyle osteosynthesis by one plain plate due to the usage of two parallel plain plates affecting a superior biomechanical result, double-plain plating [8] is not the gold standard, as used to be the case [9]. According to Meyer [10], the best biomechanical technique is the application of two plain plates along the arrangement of the compression and strain lines in the condylar region of the mandible (i.e., offset pattern). However, Aquilina et al. [9] showed with finite element analysis that a parallel orientation of two miniplates resulted in lower stresses and displacements than the use of two miniplates in an offset pattern. The same team confirmed that the use of two parallel 2.0 titanium miniplates gave a more stable configuration with lower displacements over the use of a single miniplate. The authors of this work, unable to resolve these dilemmas, adopted, after Meyer [10], the technique of laying two straight plates according to the physiologic strain lines in the bone as the gold standard and refence osteosynthesis.

The situation is similar for dedicated plates. Let us take as an example the relatively new delta plates: designs 5 and 26 [4]. On one hand, they are recommended as having high primary stability and decreased likelihood of screw loosening in experiments on animal bone [8], but the same plates in numerical experiment finite elements [11] are suspected of screw slippage from compression holes in the plate. This is why it is worth comparing all available plate designs in one experimental model.

The aim of this study was to compare any dedicated plates possible for use in ORIF of low-neck condylar fractures of the mandible.

## 2. Materials and Methods

### 2.1. Mandibles

The classification of a mandibular condylar fracture based on oblique lines was utilized in designing this study due to its proper relationship to the fracture lines observed clinically [12]. According to this classification, mandibular models were made to demonstrate low-neck condylar fractures.

**Figure 1:**
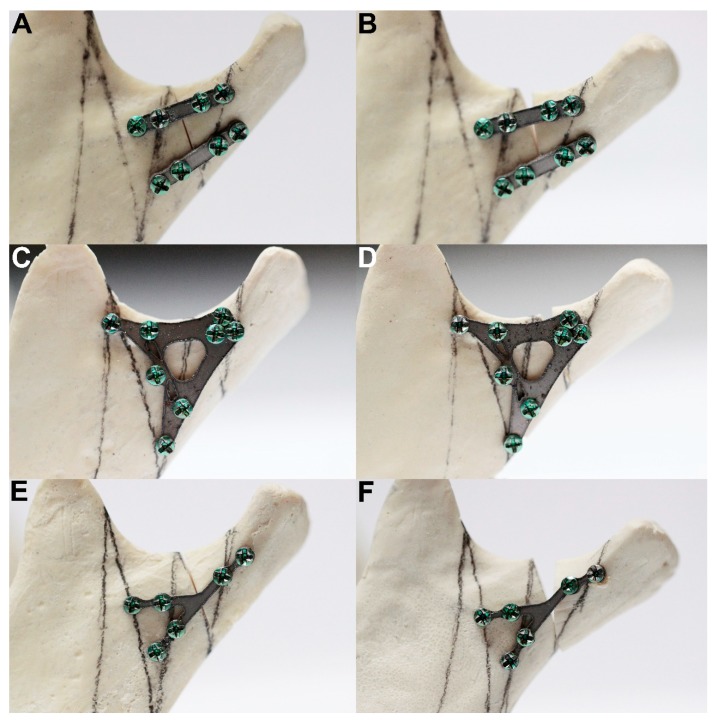
Example of the test results of double-palate fixation (**A**: design 20), rigid plate fixation (**C**: design 18) and weak plate fixation (**E**: design 28). Pictures (**B**,**D**,**F**) show the final condylar positions at the end of the loading test. Note the fracture line widths after the test illustrations: (B, D, and F). Maximal load-bearing for the double plate was 32.7 N, for design 18 it was 15.9 N, and for design 28 it was 7.1 N. The force required for 1-millimeter displacement of the fracture line for the double plate was 15.2 ± 3.5 N (reference osteosynthesis, gold standard), for design 18 it was 14.2 ± 1.3 N (rigid osteosynthesis), and for design 28 it was 5.8 ± 1.4 N (weak osteosynthesis). The differences among the plate designs were significant (*p* < 0.001).

A mandibular model made of solid foam was used in the article (Figure 1). Biomechanical testing outcomes depend on the different densities and elastic moduli of bone [13,14]. In the literature, polyurethane mandibles have been proven to be the material of choice in orthopedic implant testing, especially in fractures, and have been confirmed by the American Society for Testing and Materials [15,16]. The most natural would appear to be cadaver bone, but these differ from each other, so the results of biomechanical fatigue tests cannot be standardized [14]. Solid polyurethane material has properties comparable to those of human cancellous bone, and it is widely used as an ideal medium to mimic human cancellous bone. In our study, polyurethane mandibles (Sawbones, Vashon, WA, USA: density 0.16 g/cc, compression modulus 58 MPa) were utilized as models for fatigued mechanical tests [17,18,19,20].

### 2.2. Plates

Among the 30 available 2.0 system plates for rigid fixation of the condylar process of the mandible, only 16 designs could be applied for low-neck condylar fixation due to the anatomical structure of the neck of the mandible. Some of the other plates [4] could be used; however, manual bending would be necessary and would change the physical properties of the osteosynthesis. Therefore, only 16 plates were included in this study (Table 1). The plates were laser cut from medically certified titanium sheets (alloy grade 23, one millimeter thick).

The mandibular condyle was cut at the level of a typical low-neck fracture in the model, according to the newest classification of the fractures [12]. Proximal (i.e., condylar) and distal (i.e., ramus) fracture segments were fixed with a plate and the same 6-mm length self-tapping titanium screws of the 2.0 system. A drill bit 1.5 mm in width was used before filling the plate holes with screws. Drilling was performed perpendicular to the plate surface. Each of the seven mandibles for separate plate designs were included in one of 16 groups (112 mandibles were utilized).

### 2.3. Simulation Set

Forces on the temporomandibular joint were simulated according to the literature [4,5,21]. At 15° inferior in the sagittal plane and 10° lateral in the coronal plane, mandibles were solidly stabilized by screws on the individual base plate [4,5]. The plate was 1 mm thick, made with stainless steel, and screwed on a 0.7 m × 0.6 m tilted block with 4 × M6 holes for stabilization with bolts. In this construction, forces were generated upward, forward, and medially.

All fatigues tests were performed using a Zwick Roell Z020 universal strength machine (Zwick-Roell, Ulm, Germany). The loading force was 1 N, and the velocity of the piston was 1 millimeter per minute. All the compressive forces were pointed to the condyle. Instron software (testXpert II V3.31, Zwick Roell, Ulm, Germany) recorded the relationship between load and displacement, load for permanent deformation, and maximum load at fracture.

An irreversible change in shape was described as the starting point at which the load-displacement relationship became nonlinear. The moment when the highest load was recorded just before a sudden decrease was called the maximum load. Incidents of pull-out screws were noted for the proximal (condylar) fragment and distal (ramus) fragment of the fixation.

The plate design factor was calculated (eigenvalue equal 3.04) as statistically related to fixation rigidity, which is the main aim of ORIF and plate application [4]:

Plate Design Factor = 0.850954 × Plate height (mm) + 0.846751 × Plate width (mm) + 0.936732 × Plate surface area (mm^2^) + 0.848039 × Total fixing screws in plate, (1)

The advantage of the factor is that it only depends on the plate construction features and not on the application. It is an easy tool for future plates as an invention as well for evaluation, mainly considering their construction.

### 2.4. Statistical Analysis

Height, width, plate surface area, plate design factor, screw pull-out, and force required for one millimeter displacement of the fracture line after plate fixation were recorded for interpretation of the experimental data. The software used for statistics was Statgraphics Centurion 18 (Statgraphics Technologies Inc. The Plains City, Warrenton, VA, USA). The Kruskal–Wallis test was applied for between-design comparisons. Independent Chi^2^ tests were used to test the categorical variables. The indication of the best plate was made based on objective description. A p-value less than 0.05 was considered statistically significant.

## 3. Results

**Figure 2:**
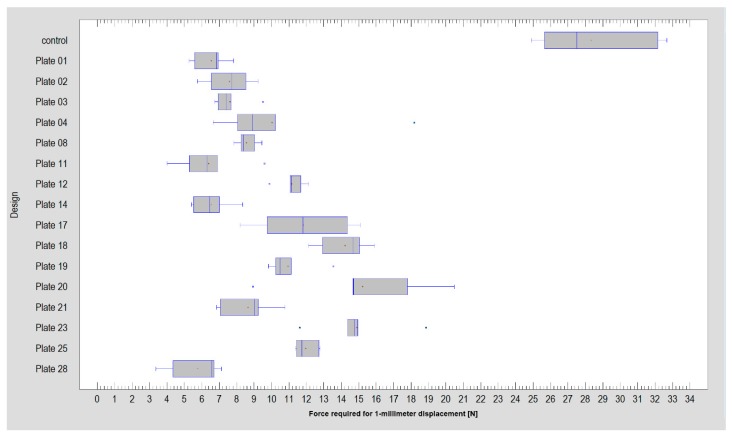
Comparison of the force required for 1-millimeter displacement of the fracture line among sixteen plate designs (including the reference of double-plate osteosynthesis, plate 20) and the intact model (i.e., control).

**Figure 3:**
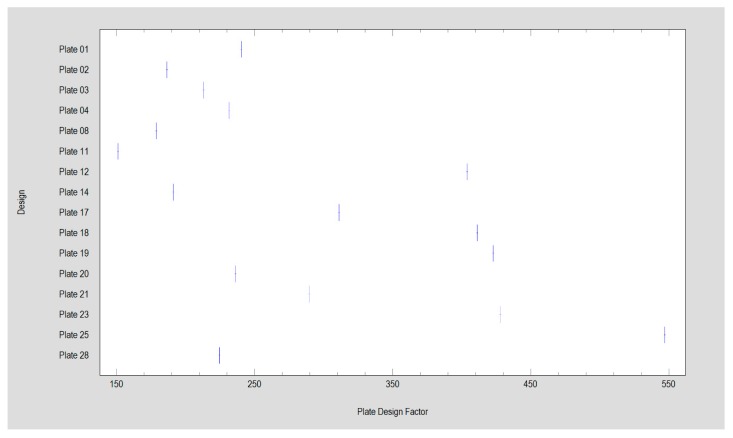
Plate design factor demonstrated by the investigated titanium plates in this study. Plate 20 is the reference, i.e., double-plain plate fixation.

**Figure 4:**
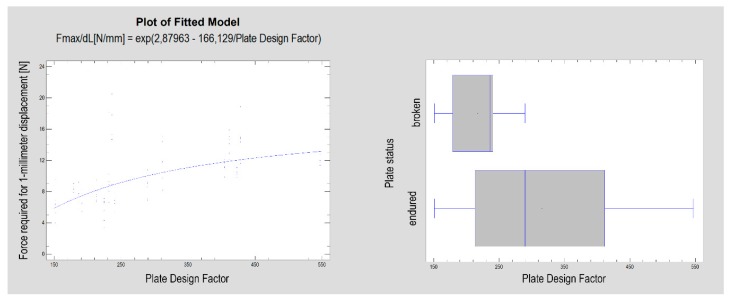
The plate design factor (according to Equation (1)) describes the construction of the plate and is related to the rigidity of the osteosynthesis performed by the dedicated plate (*p* < 0.001, left side plot). Designs with the plate design factor with a lower value were found to be more vulnerable to cracking during the test (*p* < 0.01, right side graph).

The investigated plates had four to 10 holes for screw fixation. Plates with eight, nine, and 10 holes required significantly higher forces to allow 1-millimeter displacement of the fracture line than those with fewer holes (Kruskal–Wallis test statistic = 75.68; *p* < 0.001). If it was possible to insert three screws into the condylar part (i.e., above the fracture line), the connection was more stable than with two screws (11.5 ± 2.5 N vs 7.2 ± 6.9 N; *p* < 0.001). Furthermore, four screws in the condylar part (15.2 ± 9.1 N) were more resistant than three-screw osteosynthesis (*p* < 0.001). With each screw added in the condylar part, the fixation strength increased significantly. Thereafter, the number of screws in the mandibular ramus part (i.e., distal, lower part of fixation) also influenced the stability of the whole fixation. Osteosyntheses with six and seven screws in the lower part could withstand higher loads than osteosyntheses with other numbers of screws in the ramus part: 12.7 ± 2.1 N and 11.8 ± 2.4 N, respectively (Kruskal–Wallis test statistic = 40.23; *p* < 0.001). Obviously, two-screw fixation of the lower part demonstrated the worst stability: 7.3 ± 1.4 N per 1-millimeter displacement (Figure 2, Figure 3 and Figure 4).

**Figure 5:**
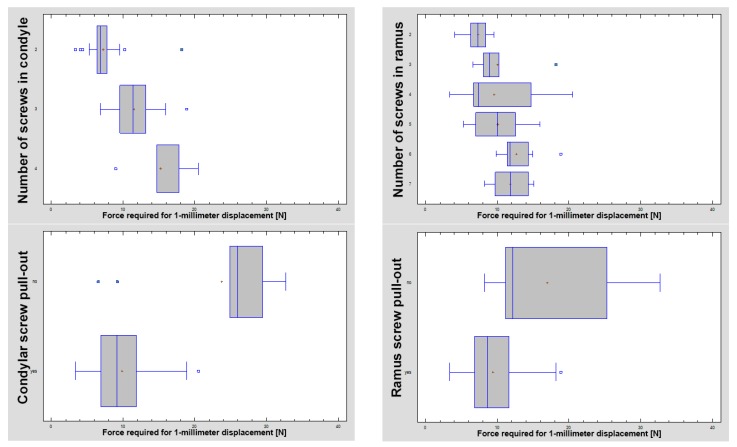
The number of screws used influenced the stability of osteosynthesis. A higher number of screws significantly improved the stability (*p* < 0.001). The trend was as follows: the more screws, the higher the force that was needed to cause one-millimeter displacement of the fracture line. The screw pull-out test revealed that most fixing screws were lost from the condylar part with a force of 9.9 ± 3.5 N versus survivor screws that resisted at a force of 23.8 ± 9.4 N and screws from the ramus part at a force of 9.4 ± 3.4 N versus 17.0 ± 8.0 N (both tests with a *p* < 0.001 significance).

Screw pull-outs were observed in the condylar fragment at a force of 9.9 ± 3.5 N versus surviving screw fixations, which reached as high as 23.8 ± 9.4 N (Kruskal–Wallis test statistic = 12.16; *p* < 0.001). The same results were found for ramus screws (Figure 5): 9.4 ± 3.4 N versus 17.0 ± 8.0 N (Kruskal–Wallis test statistic = 25.43; *p* < 0.001). A total of 85 of the 112 tested plates passed the test, bearing a load of 10.3 ± 3.1 N, as opposed to 27 cracked plates that broke at a force of 8.7 ± 4.4 N (Kruskal–Wallis test statistic = 9.20; *p* < 0.01). Moreover, it was observed (simple regression) that the displacement force of the fixed fracture depended on the plate surface (R^2^ = 43%; correlation coefficient CC = 0.66; *p* < 0.001). This was a moderately strong relationship (correlation coefficient = 0.66), as described by the following equation:

(2) $$F=e^{2.877-\frac{154.384}{Plate\;surface\;area}}$$

**Figure 6:**
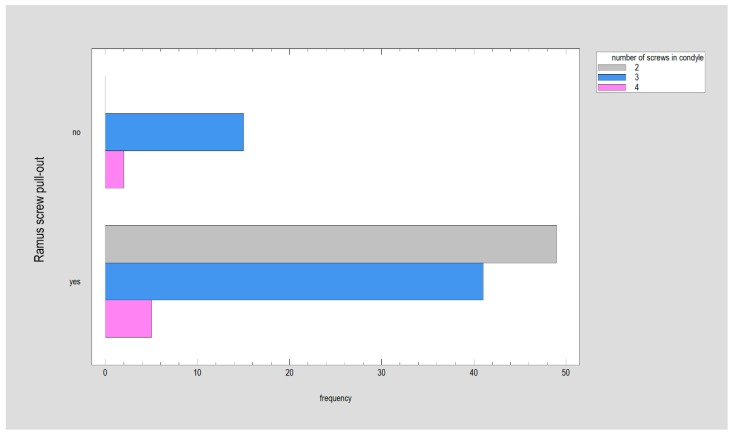
The occurrence of the loss of screws from the ramus part was related to the number of screws used in the condylar part: the highest frequency of screw pull-out from the ramus was observed as only two screws fixed the plate to the condyle of the mandible (*p* < 0.001).

Almost all plates lost their screws from the condylar part (Chi^2^ independence test = 98.57; *p* < 0.001). The only exception was design 02, in which the screws were maintained in two of seven test rounds. In the ramus part (the lower part of osteosynthesis), the situation was different; in many plates, the screws were observed at the end of the test. This situation was related to the plate design (Chi^2^ independence test = 95.93; *p* < 0.001). All screws remained in plate 12, and many screws remained in plates 17, 18, and 20. The occurrence of the loss of screws from the ramus part was related to the number of screws used in the condylar part (Figure 6; Chi^2^ independence test = 15.60; *p* < 0.001).

There was a statistically significant relationship between the force required for 1-millimeter displacement and the plate surface area (see Equation (2); *p* < 0.001) as well its derived measure: the plate design factor (R^2^ = 41%; CC = 64; *p* < 0.001). The correlation coefficient was equal to 0.64, indicating a moderately strong relationship between the variables. Moreover, as seen in Figure 4, the plate design factor was significantly higher in plates that had remained unbroken in the test (Kruskal–Wallis test statistic = 8.60; *p* < 0.01)

**Figure 7:**
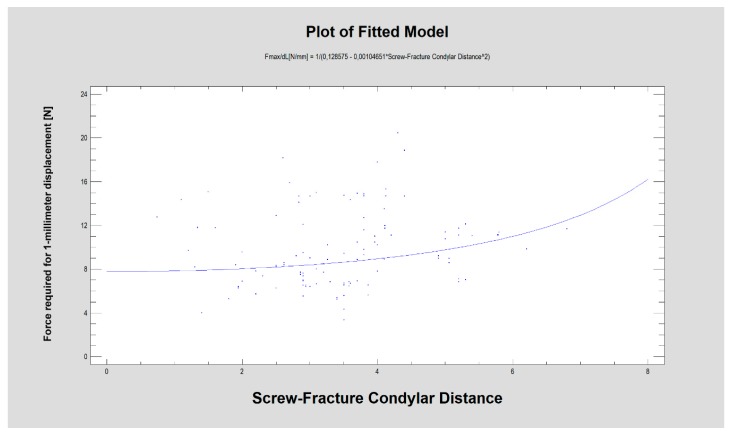
The force required for 1-millimeter displacement [N] was related to the distance [mm] between the fracture line and the nearest screw located in the condylar fragment of the mandible (*p* < 0.05).

**Figure 8:**
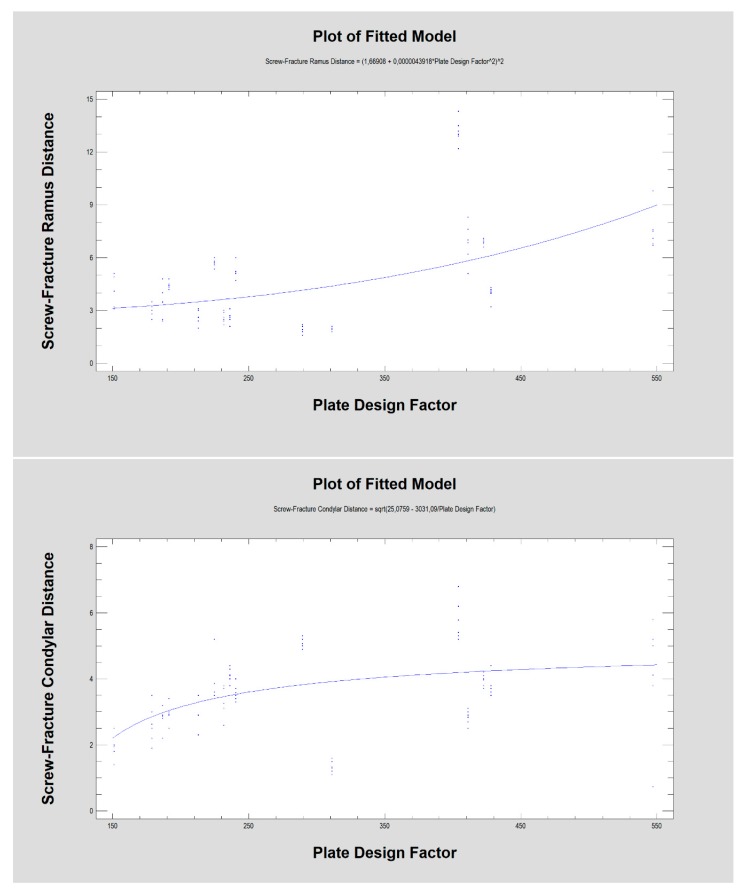
The distance from the fracture line to the nearest screw [mm], as in the ramus fragment as well as in the condylar fragment, was related to the plate design factor (in both *p* < 0.001).

As the nearest screw distance to the fracture line was considered, the average nearest distance was 4.7 ± 2.8 mm in the ramus fragment (distal) and 3.4 ± 1.2mm in the condylar fragment [proximal]. This distance in the condylar fragment was weakly related to the force required for 1-millimeter displacement (Figure 7; R^2^ = 4.5%; CC = 0.21; *p* < 0.05). No such relationship was found in the ramus fragment. There were no relationships between the screw-fracture line distance (in the condyle or ramus) and screw pull-out (condyle or ramus). The same lack of relationship was observed between this screw distance (in the condyle or ramus) and plate break. However, a moderately strong relationship was noted between the screw-fracture line distance in the ramus fragment and the plate design factor (Figure 8; R^2^ = 32%; CC = 0.57; *p* < 0.001), and when considering the screw distance in the condylar fragment, there was a weak relationship with the plate design factor (R^2^ = 23%; CC = 0.48; *p* < 0.001).

## 4. Discussion

The choice of plates dedicated anatomically for low-neck fractures is an important issue that the authors decided to resolve. This means that 14 of the 30 plates that could have been used were excluded from the comparison, due to the need of a mechanical fitting to the bone surface shape (intrasurgical or presurgical bending in the patient-specific 3D stereolithographic model). Additional experiments were also needed to prove whether other available plates could be strong enough after bending to endure mastication forces during the healing process. Without pre-bending, many of the presented plate designs were not feasible for use because they were too large for low-neck condylar process fractures. The rigidity of plates is of paramount importance for every inventor, manufacturer, user, or surgeon in ORIF. Thus, 16 plates remained for comparison.

The first observation is that low-neck fracture fixation can be performed by many available plates with high rigidity and is much more stable than that possible in high-neck fractures [5]. Regarding the degree of the surgical complications of osteosynthesis and the number of available large plates, the series of relevant fixing materials for low-neck fractures is quite long. In contrast to high-neck fracture fixation [5], in low-neck fracture fixation, the double-plain plates are not the best plates (Figure 3), but are still suitable in terms of the construction features. A series of dedicated strong plate designs can be pointed out according to the plate design factor: designs 12, 17, 18, 19, 23, and 25. These were the same code numbers as in the basal condylar fracture comparison [4]. The construction described by the plate design factor of over approximately 300 was the most resistant to screw pull-out as well displacing force [4]. However (Figure 2, Table 1), if the 1-millimeter displacing force was considered experimentally, then the double-plate again became the best solution for the osteosynthesis of low-neck fractures. Rigid fixation was reached with the use of plates 12, 17, 18, 19, 20, 23, and 25 (*p* < 0.001). This means that seven of 16 plate designs can be recommended for low-neck fixation, remembering that the generally available dedicated plates for condylar fractures account for as many as 30 types [4]. These plates are named ACPs in three versions [22], XCPs in two versions, and Auto Repositioning Plates in two versions [4,23].

Notably, the nearest screw distance to the fracture line in the condylar fragment (Figure 7) was related to the force required for 1-millimeter displacement (*p* < 0.05), which is probably a result of plate construction (Figure 8). In small plates, unfavorably, the screws must be located very closely to the fracture line in contrast to larger plates, where screws are more numerous and are often set further away from the fracture line (*p* < 0.001).

The plate recognized here as mechanically proper usually requires a wider surgical approach due to its surface area. However, big plate 17 has been specifically designed for a small transoral approach with endoscopic assistance [23]. Thus, even relatively large plates can be used via an intraoral surgical approach. Next, as the plate allowed the insertion of 8–10 screws, the fixation was stronger than in plates allowing the insertion of 4–7 screws (*p* < 0.001). Additionally, with each screw added in the condylar part, the fixation strength increased significantly (*p* < 0.001). It was only a matter of clinical possibility to apply four screws in the proximal bone fragment (condylar part). The above-mentioned statistical observation was only the confirmation of the gold standard of mandible condylar osteosynthesis by double-straight plates, which makes it possible to fit four screws. Two straight plates (design 20) fixing the bone along the stress lines in the condylar region of the mandible lead to very rigid internal fixations [9,21,22,24,25,26,27,28,29]. Obviously [22,30,31], it is not always anatomically possible to insert four screws and two plates; then, a dedicated single plate with three screws in the condylar part can be used (design 17, 18, 19, 23, or 25) with only a slight loss of osteosynthesis stability (Table 1). Anatomical problems in finding space for screws disappear in the mandibular ramus (lower part of osteosynthesis). The osteosynthesis was much more stable when six or seven screws (designs 12, 17, 23 or 25) were inserted here to fix the plate (*p* < 0.001). The selection of a plate can be based on the information [28] that its cracks occur through holes and not through bridge reinforcements. Such reinforced plates were designated 12, 23, 25, and possibly 18 and 19 (but the latter possessed only five holes in the lower part for mandibular ramus stabilization). It is worth noting that in these tests, the stability of the screws in the condylar part (*p* < 0.001) was found until the end of the test in plate designs 12, 17, 18, and 20. An interesting observation was the phenomenon that fewer screws were lost in the lower part of the fixation (ramus fragment), if the plate was attached to the condyle (upper part) by three screws (Figure 6). On the other hand, the stability of the ramus screws was often lost when there were two fixing screws in the condyle (*p* < 0.001).

Until now, there have not been such broad comparisons of plates [9,21,22,24,26,28,31], but a limitation of this study should be explained. Despite the fact that the mechanical properties of the foam models were comparable to those of real mandibles, some discrepancies in the structure of the materials were observed. For example, artificial models had an almost homogeneous pore size, whereas human mandibles consisted of a complex texture filled with differing pore sizes, which might play a role in the compression efficacy and torque of the screws. The outcomes of our research were made on a single-density foam polyurethane bone, but the biomechanical properties of the screws change with the bone density environment [19]. Further fatigue tests and finite element analysis with the changing shapes of other plates are necessary.

While the management of condylar fractures has been extensively studied and reported [9,21,22,24,25,26,28,31], there remains no consensus on what the best treatment method should include [32]. Continued debate on the optimal management methods may stem from the heterogeneity of published studies [33]. ORIF with the use of plates is likely to be the best approach for significantly displaced fractures, for patients who cannot tolerate mandible-maxillary immobilization for six weeks, and for those who want a faster return to movement of the jaw.

Currently, this study, together with two previous articles [4,5], covered the whole variability of condylar fractures according to actual classification [12]. This published series points to the one standard classification system for condylar fractures; even when fractures are divided into subgroups, they can easily be compared across studies. Last but not least, the most proper fixing plate was indicated for each of three levels of mandible condylar fractures.

## 5. Conclusions

It is advisable to consider mechanical advantages when one decides which plate to choose for ORIF in low-neck condylar fractures or only to be aware of the significant differences in mobility within the fracture line after fixation with different dedicated plates.

## Figures and Tables

**Table 1:** The tested designs of plates dedicated for osteosynthesis of low-neck condylar fractures of the mandible differed significantly (*p* < 0.001) as far as the force required for 1-millimeter displacement of the fracture line after osteosynthesis. It is the measure of stability of rigid fixation. Green cells indicate the best mechanical designs (the highest force required for 1-millimeter displacement in the fracture line after osteosynthesis). Red cells indicate the worst mechanical designs (the lowest force required for 1-millimeter displacement of the fracture line after osteosynthesis).

Design Code	Manufacturer of Similar Plate	Design View	Height [mm]	Width [mm]	Surface Area [mm^2^]	Number of Screws in Condyle	Number of Screws in Ramus	Force Required for 1-millimeter Displacement [N]
Plate 20	any	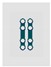	16.5	3.4	227	4	4	15.2 ± 3.5
Plate 03	Global D	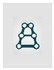	13.5	11.7	199	2	4	7.6 ± 0.9
Plate 11	Synthes	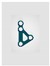	13.5	8	138	2	2	6.4 ± 1.7
Plate 08	ChM	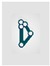	14.9	8.1	165	3	2	8.6 ± 0.5
Plate 02	Medartis	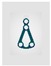	15.4	8.8	174	2	2	7.6 ± 1.2
Plate 14	Medartis	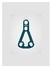	15.3	8.8	179	2	2	6.5 ± 1.0
Plate 04	Synthes	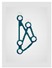	19	9.6	217	2	3	10.0 ± 3.8
Plate 21	KLS Martin	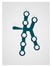	22.7	11	271	3	5	8.6 ± 1.4
Plate 12	ChM	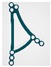	37	21	371	3	6	11.2 ± 0.7
Plate 01	Synthes	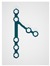	25.6	13	219	2	5	6.5 ± 0.9
Plate 28	Medicon	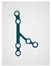	23.6	11.1	203	2	4	5.8 ± 1.4
Plate 17	KLS Martin	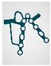	21.6	15.3	290	3	7	11.8 ± 2.4
Plate 25	ChM	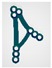	26	16.3	538	3	6	12.0 ± 0.6
Plate 23	ChM	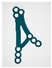	30.4	15.4	410	3	6	14.9 ± 2.1
Plate 18	UMed Lodz	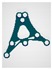	22.7	20	393	3	5	14.2 ± 1.3
Plate 19	UMed Lodz	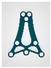	22.7	18	407	3	5	11.0 ± 1.2
